# Apoptosis of Mesothelial Cells Is Associated with the Pattern of Peritoneal Metastases in Ovarian Cancer

**DOI:** 10.3390/cancers18010102

**Published:** 2025-12-29

**Authors:** Konstantin Maksin, Magdalena Nadolna, Mateusz Wozniak, Tetiana Bocharova, Piotr Jasinski, Michal Nowicki, Ewa Nowak-Markwitz, Sebastian Szubert

**Affiliations:** 1 Department of Pathology, Clinical Hospital of Obstetrics and Gynecology, Poznan University of Medical Sciences, Polna 33 Street, 60-535 Poznan, Poland; k.maksin@ansm.pl (K.M.); pjasinski@gpsk.ump.edu.pl (P.J.); 2 Faculty of Medical Sciences, Poznan Medical University of Prince Mieszko I, 55 Bulgarska Street, 60-320 Poznan, Poland; t.bocharova@khimu.edu.ua; 3 The Student Scientific Society, Poznan University of Medical Sciences, 5 Rokietnicka Street, 60-806 Poznan, Poland; 85810@student.ump.edu.pl; 4 Student’s Research Group of Gynaecological Oncology, Poznan University of Medical Sciences, Polna 33 Street, 60-535 Poznan, Poland; 5 Division of Gynaecological Oncology, Department of Gynaecology, Poznan University of Medical Sciences, Polna 33 Street, 60-535 Poznan, Poland; mateusz.wozniak@ump.edu.pl (M.W.); ewamarkwitz@ump.edu.pl (E.N.-M.); 6 Department of Professionally Oriented Disciplines, Kharkiv International Medical University, 38 Molochna Street, 61-100 Kharkiv, Ukraine; 7 Department of Histology and Embryology, Poznan University of Medical Sciences, 6 Swiecickiego Street, 60-781 Poznan, Poland; mnowicki@ump.edu.pl; 8 Department of Gynaecological Oncology, Institute of Oncology, University of Medical Sciences, 82/84 Szamarzewskiego Street, 60-569 Poznan, Poland

**Keywords:** advanced ovarian cancer, peritoneal metastases, apoptosis, mesothelial cells

## Abstract

**Background/Objectives**: Peritoneal carcinomatosis is the leading cause of death in advanced ovarian cancer (AOC). Mesothelial cells lining the peritoneum modulate tumor implantation, yet the role of their apoptosis in metastasis development remains unclear. This study investigated the relationship between mesothelial cell apoptosis and metastatic spread in ovarian cancer (OC). **Methods**: The study included 26 patients with AOC, 11 with early-stage OC (EOC), and 13 healthy controls. Apoptotic activity in parietal peritoneal wall and omental mesothelial cells was assessed using the TUNEL technique. Metastases were classified as pushing or infiltrating. Associations with the peritoneal cancer index (PCI), BRCA mutation, and homologous recombination deficiency (HRD) status were analyzed. **Results**: Mesothelial cells adjacent to AOC metastases exhibited significantly higher apoptotic activity compared to controls (*p* < 0.05). Apoptosis was greater near infiltrating metastases than near pushing ones in both parietal (*p* < 0.01) and omental (*p* = 0.04) sites. The infiltration pattern was consistent between omental and parietal metastases (R = 0.588, *p* < 0.01). No significant differences in apoptosis were found between EOC and healthy controls, or in tumor and stromal cells between invasion types. Mesothelial apoptosis was independent of PCI, BRCA mutation, and HRD status. **Conclusions**: Our study suggests that mesothelial cell apoptosis may be associated with peritoneal spread in OC. Mesothelial cell apoptosis is more pronounced near infiltrative-type lesions, independent of BRCA/HRD status. These findings highlight mesothelial apoptosis as a relevant process in peritoneal dissemination. Further studies are needed to clarify its role in ovarian cancer progression.

## Simple Summary

Peritoneal carcinomatosis is the main cause of mortality in advanced ovarian cancer (AOC), yet the contribution of mesothelial cell apoptosis to metastatic spread remains unclear. This study analyzed apoptotic activity in parietal peritoneal wall and omental mesothelial cells from AOC patients, early-stage OC patients, and healthy controls using the TUNEL technique. Apoptosis was significantly elevated in mesothelial cells adjacent to AOC metastases in comparison to controls, particularly near infiltrating metastases. This infiltration pattern was consistent across peritoneal sites. No significant differences were observed between early-stage OC and healthy controls. Crucially, mesothelial apoptosis was independent of peritoneal cancer index, BRCA mutation, or homologous recombination deficiency status. These findings indicate that mesothelial cell apoptosis may facilitate peritoneal dissemination in ovarian cancer.

## 1. Introduction

Peritoneal carcinomatosis is the leading cause of death in advanced ovarian cancer (AOC). Additionally, peritoneal metastases are the primary contributors to morbidity and disease-related symptoms in ovarian cancer patients. Bloating, changes in bowel habits, abdominal distension, loss of appetite, and ultimately bowel obstruction are directly caused by peritoneal carcinomatosis. While most patients with advanced ovarian cancer (AOC) experience complete remission of visible lesions following cytoreductive surgery and first-line chemotherapy, many will still face a recurrence of the disease [1]. Recent advances in ovarian cancer (OC) treatment have significantly extended patients’ lives. However, most patients continue to experience progression from one therapy to another, often dealing with metastatic lesions in the peritoneal cavity [2]. A better understanding of the mechanisms behind the development of peritoneal metastasis may lead to targeted treatments for patients with advanced ovarian cancer (AOC) who are suffering from widespread neoplastic disease in this area. One area currently receiving particular attention is the process of apoptosis, which affects both the tumor cells that implant into the peritoneum and the stromal cells, including mesothelial cells. These stromal cells create a supportive environment for tumor growth and may also facilitate the invasion of cancer cells.

The positioning of metastases in advanced ovarian cancer within different areas of the peritoneal cavity is not random. In most cases of advanced ovarian cancer (AOC), metastases are predominantly found in the greater omentum. This preference for the greater omentum is influenced, in part, by the presence of “milky spots,” which are accumulations of lymphoid tissue along with a capillary network that resembles glomeruli, helping to drain peritoneal fluid [3]. However, the mechanisms of metastasis to the parietal peritoneum are not fully explained [4]. Milky spots may also contribute to partial peritoneal metastases in AOC. Some regions of the peritoneal cavity, like the gonads, mesentery, and parietal peritoneum of the posterior abdominal wall, also contain milky spots.

Conversely, the liver serosa, gastric wall, anterior abdominal wall, and small intestine wall do not contain milky spots. Animal studies have shown that in areas rich in milky spots, peritoneal metastases develop earlier than in regions that lack milky spots [5]. Analysis of resection sites in human ovarian cancer surgery also suggests that omentum and peritoneum of the posterior abdominal wall (pouch of Douglas, paracolic gutters, and diaphragm) are the most common sites of parietal peritoneal metastases [6].

The dynamics of peritoneal fluid circulation modulate the dissemination within the parietal peritoneum in AOC. Peritoneal fluid contains exfoliated cancer cells and numerous growth factors and microvesicles (exosomes, oncosomes), which are responsible for forming a metastatic niche in the peritoneum [7]. Peritoneal fluid accumulates in the peritoneal recesses, where its retention creates conditions that favor the development of metastatic deposits. These recesses are located along the posterior abdominal wall. As a result, the circulation dynamics of peritoneal fluid differ between the anterior and posterior abdominal walls. This variation may contribute to the distinct distribution patterns of peritoneal metastases that are observed in ovarian cancer.

A thin layer of mesothelial cells covers the entire abdominal cavity, including both the parietal and visceral peritoneum. These cells act as a protective barrier to prevent underlying tissue damage from various harmful factors, including peritoneal implantation of cancer cells [8]. However, by direct contact or indirectly by soluble tumor-derived factors, tumor cells may induce mesothelial cell retraction and disintegration of the protective barrier to facilitate invasion of the submesothelial area [9,10]. Recent studies suggest that cancer cells may induce phenotypic changes in the mesothelial cells. Such altered mesothelial cells are referred to as cancer-associated mesothelial (CAM) cells and may contribute to the development of peritoneal metastasis and cancer progression [11]. CAMs promote the adhesion and invasion of OC cells by regulating chemokine expression and enhancing integrin-mediated interactions with the extracellular matrix [12,13]. Au-Yeung et al. showed that the interaction of CAMs derived from intelectin-11 (ITLN1) with lactotransferrin can modulate the invasion of ovarian cancer cells in the omental tumor microenvironment [14]. OC cell-derived chemokines, such as TGF-β and HGF, can reprogram mesothelial cells into CAMs, which then amplify pro-metastatic signaling, including the production of fibronectin and the secretion of VEGF [15,16,17]. Altered mesothelial cells may also contribute to chemoresistance in ovarian cancer. Yoshihara et al. demonstrated that CAMs can induce decreased platinum sensitivity in OC cells by activating the FN1/Akt signaling pathway [18].

Therefore, several interactions exist between altered mesothelial cells and the progression of ovarian cancer. Recent studies suggest that the apoptosis of mesothelial cells can contribute to the formation of a metastatic niche and cancer progression in the peritoneal spread of cancer [4]. Apoptotic cells produce a complex myriad of autocrine and paracrine factors that interact with other cells in the microenvironment. These factors promote compensatory proliferation and tissue remodeling of the extracellular matrix. However, when these factors are released into the cancer microenvironment, they can promote tumor progression and immune escape [19,20]. Metastatic spread requires cancer cell invasion through the mesothelial layer covering peritoneal organs, which normally acts as a protective barrier. It is suggested that apoptosis of mesothelial cells may compromise this barrier, leading to the formation of gaps in the mesothelial lining that could facilitate tumor cell adhesion from malignant ascites to the submesothelial basement membrane and promote subsequent metastatic implantation [21]. The precise role of apoptotic mesothelial cells in the progression of ovarian cancer (OC) and the formation of metastases is currently unclear. Our understanding is limited to a few observations, which are often indirect or preliminary, and the underlying mechanisms have not been thoroughly explored. Therefore, the primary aim of this preliminary study was to investigate the relationship between the apoptosis of mesothelial cells and the development of peritoneal metastasis in ovarian cancer.

## 2. Materials and Methods

### 2.1. Patients and Sample Collection

The study involved two groups of patients: Group 1: 26 patients with advanced-stage ovarian cancer (FIGO IIIC) who exhibited both omental and parietal peritoneal wall spread. Group 2: 11 patients with early-stage ovarian cancer (FIGO IC) who presented with ascites. Additionally, there was a control group consisting of 13 women who underwent surgery for benign gynecological conditions, mostly due to menorrhagia.

From each patient with advanced-stage ovarian cancer (AOC, group 1), the following peritoneal specimens were collected: omentum with visible metastasis, omentum that was macroscopically and microscopically free of metastases, parietal peritoneum with a metastatic nodule, and parietal peritoneum that was macroscopically and microscopically free of metastases. A total of 52 specimens with metastases and 52 specimens without metastases were analyzed. We aimed to evaluate the apoptosis of metastasis-associated mesothelial cells (MCs near ovarian cancer metastasis) and that of MCs from the peritoneum not invaded by cancer cells.

**Figure 1:**
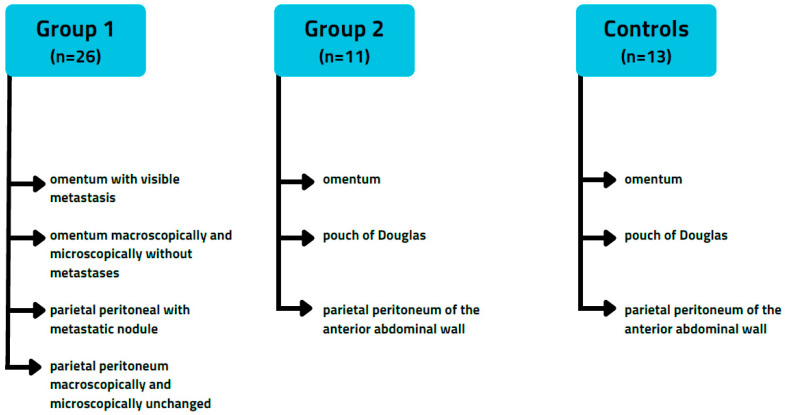
Flowchart presenting tissue samples obtained in the study. The advanced-stage ovarian cancer group included stage IIIC patients according to the International Federation of Gynecology and Obstetrics (FIGO) 2023 classification. The early-stage ovarian cancer group included patients with ovarian tumors confined to one or both ovaries, accompanied by ascites with cancer cells (stage IC). The control group included women treated due to benign gynecological conditions, such as menorrhagia.

For every early-stage ovarian cancer (EOC, group 2) patient and the control group, specimens were collected from the omentum, the pouch of Douglas, and the parietal peritoneum of the anterior abdominal wall. Therefore, in the case of early-stage OC, 11 specimens of omentum and 22 specimens of parietal peritoneum were analyzed. For the control group, we included 13 specimens of omentum and 26 specimens of parietal peritoneum. The flowchart that outlines the specimens collected during the study is displayed in Figure 1. Each specimen was excised with a cold knife to avoid tissue damage caused by scissors or electrocautery.

### 2.2. Evaluation of the Apoptosis and the Type of Metastasis

Paraffin-embedded tissue slides were prepared using the standard protocol: 1. 4% formalin immersion for 24 h at room temperature; 2. tissue dehydration in an ascending concentration of ethanol 70–90–100% 3 times each for 30 min at room temperature, followed by chloroform immersion for 2 h at 37 °C; 3. chloroform/paraffin 1:1 mixture immersion at 37 °C for 1 h; 4. paraffin embedding at 58 °C; 5. cutting of 3–5 μm slides, with subsequent water bath floating at 56 °C and mounting on histological slides.

Apoptotic cells were detected using a TUNEL Assay Kit, HRP-DAB (Abcam, Cambridge, UK; ab206386), according to the manufacturer’s instructions and as previously described [22]. Briefly, sections were deparaffinized, rehydrated, and permeabilized with proteinase K prior to incubation with terminal deoxynucleotidyl transferase (TdT) and labeled nucleotides. TUNEL labeling was visualized using an HRP-conjugated antibody and 3,3′-diaminobenzidine (DAB) as chromogen. Appropriate positive (DNase I-treated sections) and negative (TdT omission) controls were included in each staining run.

To distinguish apoptosis from necrosis, consecutive hematoxylin and eosin (H&E)–stained sections were examined in parallel. Areas showing morphological features of necrosis, including loss of nuclear detail, cytoplasmic eosinophilia, and tissue disintegration, were identified and excluded from TUNEL evaluation, even if TUNEL positivity was present.

Apoptosis was assessed using a semi-quantitative scoring system based on the percentage of TUNEL-positive mesothelial cells evaluated in at least five randomly selected high-power fields (HPFs, ×400) per case. Scores were assigned as follows: 0, no positive cells; 1, ≤4% positive cells; 2, 5–15% positive cells; and 3, >15% positive cells. Two pathologists classified each selected case independently (KM and TB). In cases of discrepant assessments, the results were reviewed jointly, and a consensus was reached.

**Figure 2:**
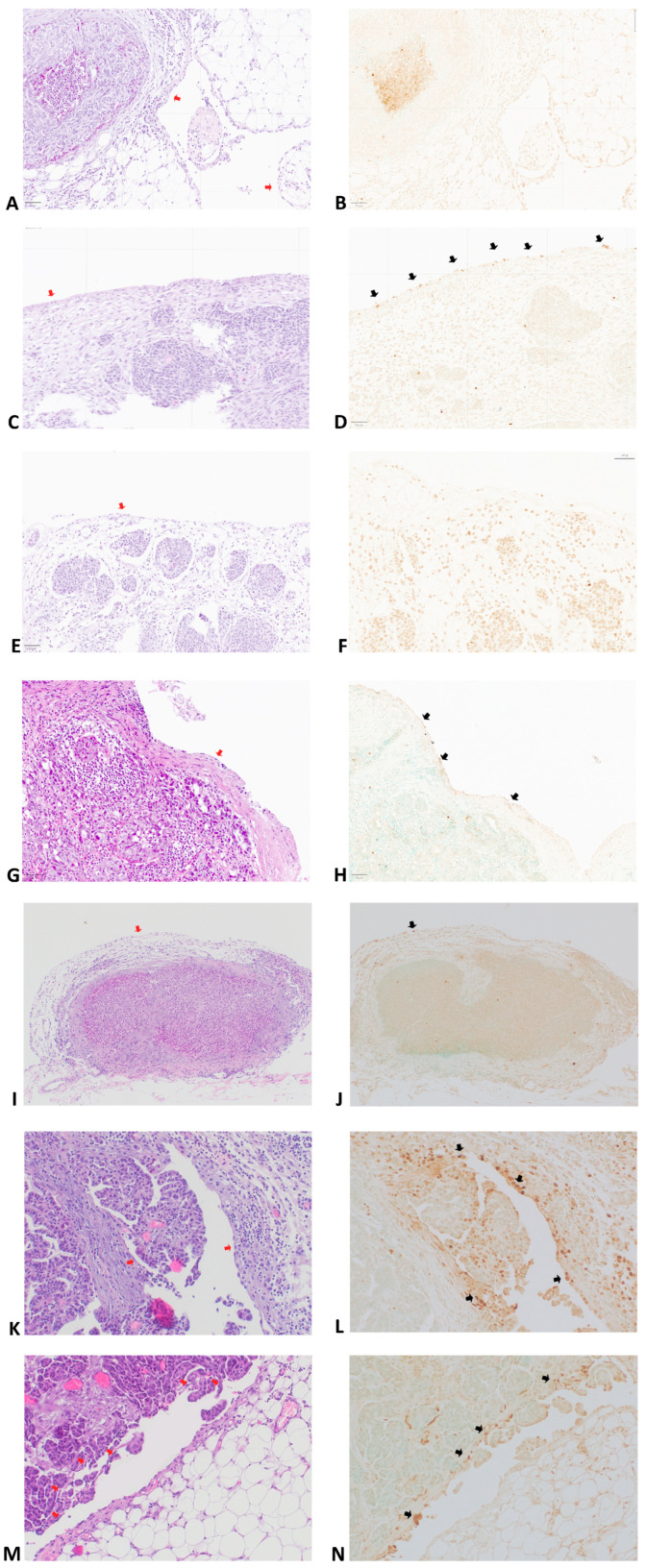
Mesothelial cells in patients with ovarian cancer. Panels (**A**,**C**,**E**,**G**,**I**,**K**,**M**) show hematoxylin and eosin (**H**,**E**) staining, and panels (**B**,**D**,**F**,**H**,**J**,**L**,**N**) show the corresponding TUNEL reactions. Red arrows show the mesothelium, while black arrows show mesothelial cells with TUNEL reaction. (**A**–**J**) Magnification ×100; (**K**–**N**) magnification ×200. (**A**,**B**) Omental metastasis of the pushing type, with absence (0) of TUNEL reactivity. (**C**,**D**) Omental metastasis of the infiltrating type, demonstrating high (3+) TUNEL reaction in the mesothelium. (**E**,**F**) Peritoneal metastasis of the pushing type, with absent (0) TUNEL reactivity. (**G**,**H**) Parietal peritoneal metastasis showing moderate (2+) TUNEL reaction within the mesothelium. (**I**,**J**) Example of pushing-type metastasis in the omentum with TUNEL reaction in a single mesothelial cell. (**K**,**L**) Infiltrative-type metastasis in the parietal peritoneum with high (3+) TUNEL reaction in the metastasis-associated mesothelial cells. (**M**,**N**) Infiltrative-type metastasis in the omentum showing a region of high (3+) TUNEL reaction in the metastasis-associated mesothelial cells.

One of the objectives of this study was to evaluate the interrelationship between apoptosis processes in CAM mesothelial cells and the type of invasion exhibited by tumors implanted into the peritoneum. Reyes et al. classified the invasive patterns of metastatic ovarian carcinoma into two principal categories: *pushing* and *infiltrating* [23]. Tumors with a pushing or circumscribed invasive pattern were characterized by a well-defined and smooth border at the tumor–stroma interface, often accompanied by a microscopically visible desmoplastic pseudocapsule formation. In contrast, tumors with an irregularly infiltrative pattern displayed jagged, finger-like, or papillary projections at the invasive front, lacking a continuous pseudocapsule. For lesions exhibiting both patterns, classification was based on the predominant growth type within the examined tissue. Representative images are presented in the Figure 2.

The extent of peritoneal dissemination in AOC patients was assessed using the peritoneal cancer index (PCI) proposed by Sugarbaker et al. [24].

In the AOC group, we performed multivariate survival analysis to investigated the impact of the following confounders on patient survival: primary (PDS) or interval debulking surgery (IDS), complete macroscopic resection or macroscopic residual disease, presence or absence of BRCA1/2 mutation, HRD or HRP, pushing or infiltrating type of metastasis in the omentum, pushing or infiltrating type of metastasis in the parietal peritoneum, TUNEL staining in metastasis-associated mesothelial cells in the parietal peritoneum, TUNEL staining in metastasis-associated mesothelial cells in the omentum.

### 2.3. Statistical Analysis

Nonparametric tests were used for statistical comparisons. The Mann–Whitney U test was applied for two-group comparisons, and the Kruskal–Wallis test was used for analyses involving three or more groups. Correlations were assessed using Spearman’s rank correlation coefficient (rho). For 2 × 2 contingency tables, Fisher’s exact test was applied. Multivariate survival analysis was conducted using Cox proportional-hazards regression with the stepwise entering method. Statistical analysis was conducted using MedCalc 11.4.2.0, MedCalc Software, Seoul, Republic of Korea, and GraphPad In Stat 3.06, GraphPad Software Inc., San Diego, CA, USA.

### 2.4. Bioethics Committee

This study received Poznan University of Medical Sciences Ethical Committee approval (946/22 and 803/22), and all patients gave written informed consent.

## 3. Results

**Figure 3:**
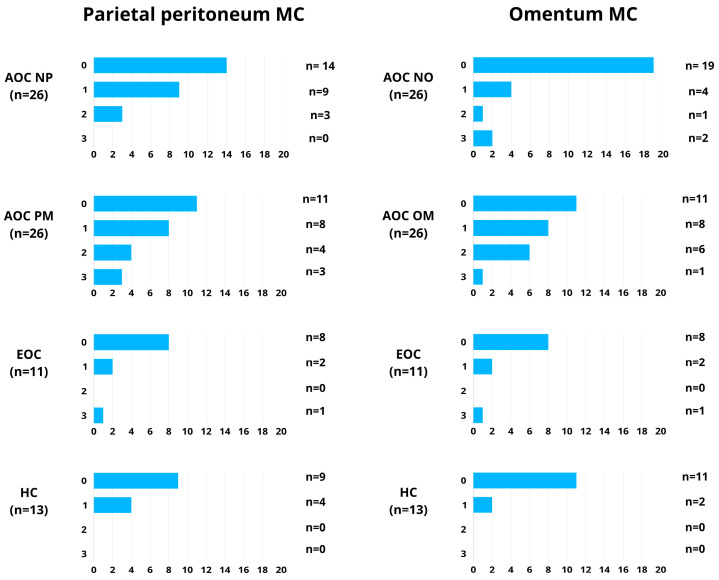
Distribution of specimens according to TUNEL staining scores (0–3) in mesothelial cells (MC) of the parietal peritoneum and omentum across the analyzed groups. Bar charts present the number of specimens assigned to each TUNEL score (0, 1, 2, and 3) in the examined tissues. AOC—advanced-stage ovarian cancer; NP—macroscopically and microscopically normal peritoneum without cancer infiltration; MP—peritoneal metastasis; EOC—early-stage ovarian cancer; HC—healthy controls; NO—macroscopically and microscopically normal omentum without cancer infiltration; OM—omental metastasis. Metastasis-associated MC refers to mesothelial cells adjacent to peritoneal or omental metastases. The total number of specimens analyzed in each group is indicated (n).

Figure 3 demonstrates the distribution of specimens according to TUNEL staining scores (0–3) in mesothelial cells of the omentum and parietal peritoneum across the analyzed groups.

**Figure 4:**
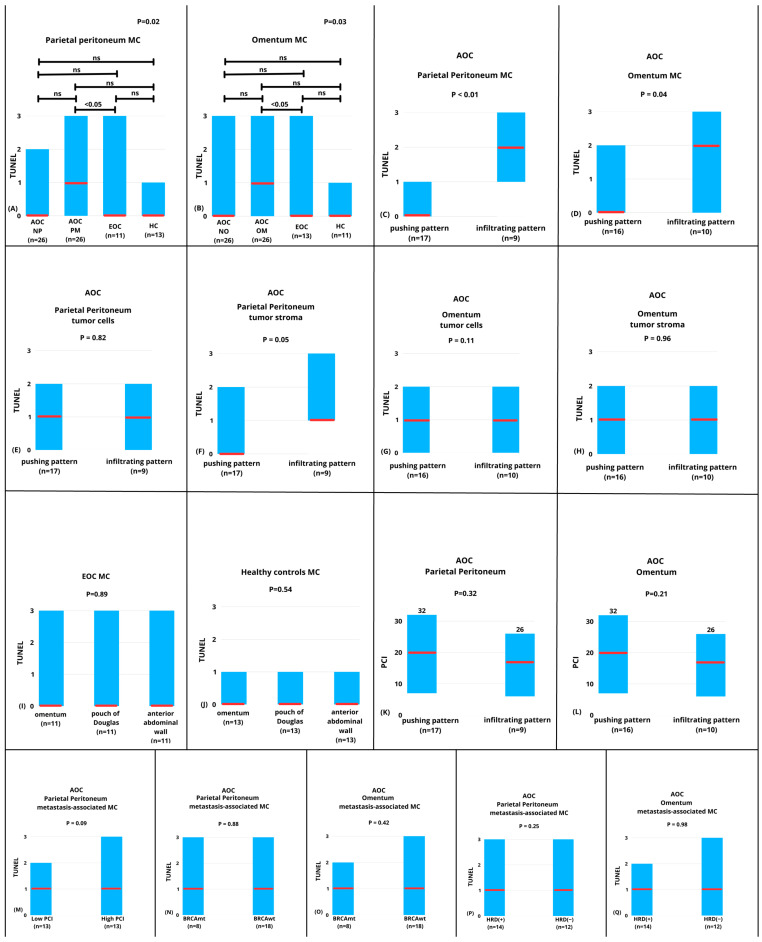
TUNEL staining in mesothelial cells (MCs) from parietal peritoneum and omentum in ovarian cancer and healthy controls. TUNEL—terminal deoxynucleotidyl transferase (TdT)-mediated dUTP nick-end labeling; AOC—advanced-stage ovarian cancer; NP—macroscopically and microscopically normal peritoneum without cancer infiltration; MP—peritoneal metastasis; EOC—early-stage ovarian cancer; HC—healthy controls; NO—macroscopically and microscopically normal omentum without cancer infiltration, OM—omental metastasis; PCI—peritoneal cancer index; metastasis-associated MC—MCs near peritoneal or omental metastasis. BRCAmt—BRCA-mutated ovarian cancer; BRCAwt—BRCA wild-type ovarian cancer; HRD (+)—homologous recombination-deficient ovarian cancer; HRD (−)—homologous recombination-proficient ovarian cancer. (**A**) Significant differences in parietal peritoneum MCs (*p* = 0.02); post-hoc significance only between AOC metastasis-associated MCs and healthy controls. (**B**) Significant differences in omental MCs (*p* = 0.03); post-hoc significance only between AOC metastasis-associated MCs and healthy controls. (**C**,**D**) Higher TUNEL staining in metastasis-associated MCs adjacent to infiltrating vs. pushing metastases in parietal peritoneum ((**C**), *p* < 0.01) and omentum ((**D**), *p* = 0.04). (**E**–**H**) No differences in TUNEL staining in tumor cells or in tumor stroma between pushing and infiltrating patterns of metastasis. (**I**,**J**) No site-related differences in the TUNEL staining in EOC patients ((**I**), *p* = 0.89) or healthy controls ((**J**), *p* = 0.54). (**K**,**L**) PCI did not differ between pushing and infiltrating metastases in parietal peritoneum ((**K**), *p* = 0.32) or omentum ((**L**), *p* = 0.21). (**M**) No difference in TUNEL staining in metastasis-associated MCs between patients with low and high PCI (*p* = 0.09). (**N**,**O**) BRCA status is not associated with MC TUNEL staining in the parietal peritoneum ((**N**), *p* = 0.88) or omentum ((**O**), *p* = 0.42). (**P**,**Q**) HRD status is not associated with MC TUNEL staining in the parietal peritoneum ((**P**), *p* = 0.25) or omentum ((**Q**), *p* = 0.98).

We observed a significant difference in median TUNEL staining in parietal peritoneum mesothelial cells (PPMCs) between AOC normal peritoneum (AOC NP), AOC with peritoneal metastasis (AOC PM), EOC, and healthy controls (*p* = 0.02). However, when post-hoc tests were performed, a significant difference was found only between metastasis-associated PPMCs in AOC (AOC PM) and PPMCs from healthy controls (*p* < 0.05, Figure 4A).

Similarly, we observed a significant difference in median TUNEL staining in omental mesothelial cells (OMCs) between AOC normal peritoneum (AOC NP), AOC with peritoneal metastasis (AOC), EOC, and healthy controls (*p* = 0.03). Similarly, when post-hoc tests were performed, a significant difference was found only between metastasis-associated OMCs (AOC OM) in AOC and OMCs from healthy controls (*p* < 0.05, Figure 4B).

Parietal peritoneal metastases in AOC presented a pushing pattern in 17 cases (17/26) and an infiltrating pattern in 9 cases (9/26). In the case of omental metastases, the pushing and infiltrating pattern was presented in 16 and 10 cases, respectively. Both in the case of PPMCs (*p* < 0.01) and OMCs (*p* = 0.04), median TUNEL staining was significantly higher in mesothelial cells near metastases presenting an infiltrating pattern compared to those presenting a pushing pattern (Figure 4C and Figure 4D, respectively). We found a significant, positive correlation between the type of infiltration between parietal peritoneum and omental metastases (R Spearman = 0.588, *p* < 0.01). Twenty-two of the analyzed patients presented the same type of infiltration in the parietal peritoneum and omental metastases. In four patients (15%), a different type of infiltration was observed in the parietal peritoneum and omentum. In three patients, an infiltrating pattern of metastases was observed in the omental metastases, whereas a pushing pattern was noted in the parietal peritoneum. On the other hand, one patient was diagnosed with pushing metastases within the omental sample while having infiltrating metastases in the parietal peritoneum.

One of our aims was to evaluate specimens of the peritoneum not involving cancer in AOC patients. Therefore, we took a sample of omentum and parietal peritoneum that showed no macroscopic involvement. However, in the case of five patients (19%), some of the macroscopically unchanged specimens of the peritoneum were found to involve cancer under microscopic evaluation. These patients had infiltrating metastases in both the omental samples with visible metastasis (4 cases) and the parietal peritoneal samples with metastatic nodules (2 cases). However, in the case of macroscopically normal but microscopically involved peritoneum specimens, the microscopic evaluation indicated pushing-type metastases.

We found no difference in median TUNEL staining in parietal peritoneal metastases in tumor cells (*p* = 0.82, Figure 4E) or in the tumor stroma (*p* = 0.05, Figure 4F) between pushing and infiltrating metastases. Similarly, there was no difference in median TUNEL staining between omental metastases in tumor cells (*p* = 0.11, Figure 4G) or in the tumor stroma (*p* = 0.96, Figure 4H) between pushing and infiltrating metastatic patterns.

We found no difference in median TUNEL staining in the MCs from the omentum, pouch of Douglas, or parietal peritoneum of the anterior abdominal wall in the group of EOC patients. (*p* = 0.89, Figure 4I). Similarly, there was no difference in the median TUNEL staining in MCs from the omentum, the pouch of Douglas, or the parietal peritoneum of the anterior abdominal wall in the healthy controls (*p* = 0.54, Figure 4J)

The median PCI in the AOC group was 18 (range, 6–32). There was no difference in median PCI index between patients with pushing and infiltrating metastases within the parietal peritoneum in the AOC group (median 21, range 8–32 vs. 17, range 6–26; *p* = 0.32, Figure 4K). Similarly, there was no difference in median PCI between patients presenting with pushing and infiltrating metastases in the omentum in the AOC group (median 20, range 7–32 vs. 17, range 6–26; *p* = 0.21, Figure 4L). Furthermore, there was no statistically significant difference in median TUNEL staining in parietal peritoneal metastasis-associated MC metastasis between patients with low (below median) and high PCI (equal or above median, *p* = 0.09, Figure 4M).

In the group of AOC, eight women were diagnosed with BRCA-mutated ovarian cancer, 14 (53%) women with homologous recombination deficiency (HRD-positive), and 12 (47%) women without homologous recombination deficiency (HRD-negative). We found no difference in the median TUNEL staining in metastasis-associated MCs depending on BRCA mutation status in either the parietal peritoneum (*p* = 0.88) or the omentum (*p* = 0.42) (Figure 4N and Figure 4O, respectively). Similarly, there was no difference in median TUNEL staining in metastasis-associated MCs depending on HRD status in either the parietal peritoneum (*p* = 0.25) or the omentum (*p* = 0.98) (Figure 4P and Figure 4Q, respectively). There were no differences in the type of infiltration in metastatic lesions depending on BRCA mutation and HRD status (Table 1).

In the multivariate survival analysis, we found that the presence of macroscopic residual disease (hazard ratio (HR) = 4.4; 95% CI = 1.46–13.38) and IDS (HR = 7.19; 95% CI = 1.30–39.56) were independently associated with shortened overall survival. The presence or absence of BRCA1/2 mutation, HRD or HRP, pushing or infiltrating type of metastasis in the omentum, pushing or infiltrating type of metastasis in the parietal peritoneum, TUNEL staining intensity in metastasis-associated mesothelial cells in the parietal peritoneum, and TUNEL staining intensity in metastasis-associated mesothelial cells in the omentum were not related to patient OS.

The complete dataset of the analyzed samples is presented in Supplementary Table S1 (available online at Zenodo [DOI 10.5281/zenodo.18016552]) [25].

## 4. Discussion

This study found that mesothelial cells surrounding parietal peritoneum and omental metastases in advanced ovarian cancer (AOC) exhibited significantly higher median TUNEL staining compared to normal peritoneal cells from healthy controls. TUNEL staining is commonly used as a marker for apoptosis, indicating that apoptotic mesothelial cells play a role in the development of peritoneal carcinomatosis associated with ovarian cancer.

Only a limited number of studies have explored the relationship between mesothelial cell apoptosis and the development of metastasis within the abdominal cavity. For instance, Yokoi et al. demonstrated that the intraperitoneal injection of cancer-derived extracellular vesicles (EVs) containing MMP1 mRNA into the peritoneal cavity of mice resulted in apoptotic cell death of mesothelial cells, as confirmed by TUNEL staining and scanning electron microscopy. This apoptotic response in mesothelial cells was subsequently linked to the progression of cancer with peritoneal dissemination in the mice [4]. The authors also found that human peritoneal mesothelial cells cultured with OC EVs induce mesothelial cell apoptosis that develops gaps between cells, called “open areas,” that could enable cancer invasion [4]. In a study by Steitz et al., the authors showed that cancer-associated natural killer (NK) cells induce TRAIL-dependent apoptosis of human mesothelial cells in OC patients [26].

Furthermore, the authors demonstrated a significantly increased matrix invasion in three-dimensional culture by tumor cells when the mesothelial cell monolayer was disrupted by TRAIL-mediated apoptosis. In the same study, the authors found areas of apoptotic mesothelial cells in omentum specimens and apoptotic mesothelial cells in ascites samples from OC patients with omental micrometastases. Steitz et al. suggested that their observations support the hypothesis that the initial stages of metastasis involve disruption of the mesothelium through the induction of apoptosis [26].

In our study, we investigated the presence of apoptotic mesothelial cells in AOC, EOC, and healthy individuals. In the case of EOC, we chose patients with tumors confined to one or two ovaries with the presence of ascites (stage IC). Several studies showed that a malignant ascites contains many factors that force peritoneal metastasis development in OC [27]. Therefore, we suspected higher TUNEL staining in mesothelial cells from EOC compared to healthy individuals. Our results showed no difference between EOC patients and healthy individuals. However, the lack of difference may be caused by the limited number of EOC cases. In addition, we evaluated mesothelial cells from various regions of the peritoneal cavity, which differ in their rates of metastasis development (pouch of Douglas, omentum, and anterior abdominal wall). Similarly, there were no differences in TUNEL staining in mesothelial cells.

Furthermore, we evaluated the presence of apoptotic mesothelial cells in AOC. In those cases, we evaluated peritoneal samples with and without malignant infiltration. We found higher TUNEL staining only in the mesothelial cells near peritoneal metastases. When taken together, our results suggest that mesothelial cell apoptosis is a hallmark of metastasis rather than an initial stage preceding metastasis development.

In the case of AOD, we evaluated peritoneal samples from both the parietal peritoneum and the omentum, with and without malignant infiltration. We found higher TUNEL staining only in the mesothelial cells near peritoneal metastases. The median TUNEL staining in mesothelial cells from the omentum, both macroscopically and microscopically, and the parietal peritoneum, both macroscopically and microscopically, was unchanged and did not differ from that of healthy individuals and EOC. When taken together, our results suggest that mesothelial cell apoptosis is a hallmark of developing metastasis, rather than an initial stage preceding its development.

We also compared the degree of mesothelial cell apoptosis depending on the type of infiltration in peritoneal metastasis. Our study included only high-grade serous ovarian cancers, and in the group of AOC, 53% were HRD-positive tumors, suggesting a BRCAness phenotype. Soslow et al. observed different tumor morphological features of BRCA-associated and BRCA-unassociated OC [28]. Next, Reyes et al. distinguished pushing and infiltrating growth of metastases, and found BRCA-unassociated tumors to exhibit a more common infiltrating pattern compared to BRCA-associated OC [23]. Hussein et al. also show that patients with pushing pattern metastases have significantly better clinical outcomes than patients with infiltrative metastases [29]. Our study found no difference in the median TUNEL staining depending on BRCA mutation and HRD status. However, our study group included a lower number of patients than the study by Reyes et al. [23].

On the other hand, we found significantly higher mesothelial cell apoptosis near infiltrating-type metastases compared to metastases with a pushing type of growth. The association was presented both in the parietal peritoneum and in the omentum. Therefore, in our study group, the morphological pattern of metastatic growth was associated with mesothelial cell apoptosis but not with BRCA mutation/HRD status. In the case of four AOC patients (15%), the type of tumor growth differed in parietal peritoneum and omental metastases. Furthermore, in the case of 19% of AOC patients with infiltrative metastases, microscopic evaluation of macroscopically normal peritoneum revealed small tumor metastases of pushing pattern growth. These observations suggest that the morphological pattern of metastasis is not only related to BRCA mutation/HRD status but may also be driven by mesothelial cell apoptosis, and the pushing pattern may precede the infiltrating type of metastasis.

The Peritoneal Cancer Index (PCI) quantifies tumor dissemination in the peritoneal cavity [24,30]. A high PCI is associated with a poor prognosis in many human cancers, including ovarian cancer (OC), as it reflects the extent and burden of peritoneal metastases. Our study did not find a significant association between mesothelial cell apoptosis and PCI, suggesting that the degree of tumor spread, as measured by PCI, may not directly influence the apoptotic response of mesothelial cells. Similarly, there was no observable relationship between the morphological type of metastasis—whether pushing or infiltrating type—and the PCI. These observations indicate that neither the extent of peritoneal tumor dissemination nor the morphological growth pattern of metastases is determined by PCI, but rather may be governed by intrinsic tumor biology, molecular signaling pathways, and the host immune response.

Several limitations of this study should be acknowledged. First, apoptosis of mesothelial cells was assessed solely by TUNEL staining. The lack of additional apoptosis-specific markers, such as cleaved caspase-3, cleaved poly (ADP-ribose) polymerase (PARP), or members of the Bcl-2 family, limits the ability to definitively attribute the observed TUNEL positivity to apoptotic cell death [31]. The second limitation of our study is its retrospective design and the limited number of cases, which notably reduces the value of subgroup analyses. Therefore, the results of this study should be regarded as preliminary and hypothesis-generating. Despite these limitations, our findings provide initial evidence suggesting increased mesothelial cell death in the parietal peritoneum and omentum of patients with OC, particularly in the vicinity of metastatic lesions. These observations warrant further investigation in larger, prospective studies incorporating multiple, specific markers of apoptosis to more precisely define the mechanisms of mesothelial cell injury in ovarian cancer progression.

## 5. Conclusions

The results of our study suggest that mesothelial cell apoptosis is a characteristic of established, rather than initiating, metastatic disease. Apoptosis is more pronounced near infiltrative-type metastases than in pushing-type lesions, independent of BRCA/HRD status; therefore, mesothelial cell apoptosis may contribute to the growth pattern of metastasis. These findings highlight mesothelial apoptosis as a relevant process in peritoneal dissemination. Further research is needed to clarify the potential contribution of mesothelial cell apoptosis to cancer development.

## Figures and Tables

**Table 1:** The relationship between the type of infiltration in metastatic lesions in ovarian cancer patients and BRCA mutation and homologous recombination deficiency status.

Localization	Invasive Pattern of Metastasis	BRCAmt (N)	BRCAwt (N)	*p*-Value
Omentum	Pushing	6	10	0.42
	Infiltrating	2	8	
Parietal peritoneum	Pushing	5	12	1.00
	Infiltrating	3	6	
		HRD (+)	HRD (−)	
Omentum	Pushing	10	6	0.42
	Infiltrating	4	6	
Parietal peritoneum	Pushing	11	6	0.28
	Infiltrating	3	6	

BRCAmt—BRCA-mutated ovarian cancer; BRCAwt—BRCA wild-type ovarian cancer; HRD (+)—homologous recombination-deficient ovarian cancer; HRD(−)—homologous recombination-proficient ovarian cancer. N—number of cases. The invasive patterns of metastases were classified according to Reyes et al. [23].

## Data Availability

The original data presented in the study are openly available in Zenodo Repository [https://doi.org/10.5281/zenodo.18016552].

## Supplementary Materials

The supplementary data presented in this study are openly available in Zenodo at https://doi.org/10.5281/zenodo.18016552 (22 December 2025).
